# P-1243. Contezolid Tablets for Central Nervous System Tuberculosis in Adult Patients: pharmacokinetic and blood-brain barrier penetration

**DOI:** 10.1093/ofid/ofaf695.1435

**Published:** 2026-01-11

**Authors:** Ning Li, Xuan Wang, Qihui Liu, Hailan Wu, Yuanbo Lan, Xin Yu, Lin Ye, Shengsheng Liu, Bo Duan, Yuanyuan Chen, Zhixiong Fang, Junwei Cui, Fan Xia, Jing Zhang, Wenhong Zhang

**Affiliations:** Huashan Hospital, Shanghai Medical College, Fudan University, shanghai, Shanghai, China (People's Republic); Huashan Hospital, Shanghai Medical College, Fudan University, shanghai, Shanghai, China (People's Republic); Huashan Hospital, Shanghai Medical College, Fudan University, shanghai, Shanghai, China (People's Republic); Huashan Hospital, Fudan University, Shanghai, Shanghai, China; Affiliated Hospital of ZunYi Medical University, Zunyi, Guizhou, China; The Fifth People's Hospital of Suzhou, Suzhou, Jiangsu, China; Jiangxi Chest Hospital, Nanchang, Jiangxi, China; Anhui Chest Hospital, Hefei, Anhui, China; Hunan Chest Hospital, Changsha, Hunan, China; Hangzhou Red Cross Hospital, Hangzhou, Zhejiang, China; The Central Hospital of Xiangtan, Changsha, Hunan, China; The First Affiliated Hospital of Xinxiang Medical University, Xinxiang, Henan, China; 905 Hospital of People's Liberation Army Navy, Shanghai, Shanghai, China; Huashan Hospital, Fudan University, Shanghai, Shanghai, China; Huashan Hospital, Fudan University, Shanghai, Shanghai, China

## Abstract

**Background:**

Central nervous system tuberculosis (CNS TB), including tuberculous meningitis, is a life-threatening condition with high mortality rates ^[1]^. Contezolid, a next-generation oxazolidinone antibiotic, has shown comparable efficacy to linezolid but with a significantly improved safety profile ^[2]^. This exploratory study evaluated the pharmacokinetics (PK), and blood-brain barrier penetration of contezolid tablets in seven adult patients with CNS TB.

**Figure 1:**
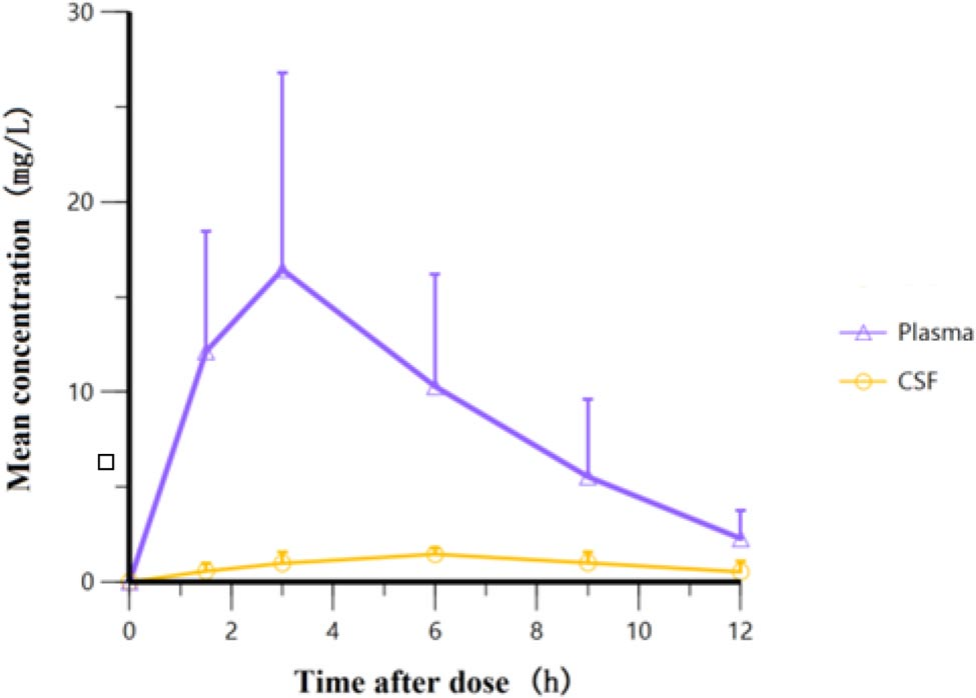
Concentration-time curves of contezolid in plasma and CSF at steady-state

**Figure 2:**
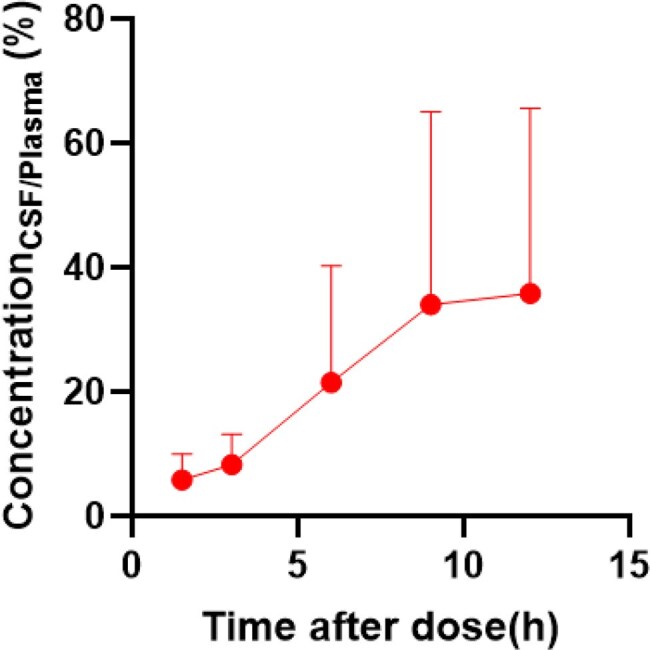
Contezolid CSF plasma concentration ratio

**Methods:**

Participants received a standardized anti-TB regimen, which includes rifampin: 10 mg/kg/day (maximum 600 mg), isoniazid: 5 mg/kg/day (maximum 600 mg), pyrazinamide: 30 mg/kg/day (maximum 2 g), levofloxacin: 500 mg/day, and dexamethasone and mannitol for adjunctive therapy. In addition to this regimen, contezolid tablets were administered at a dose of 800mg every 12 hours. Blood and cerebrospinal fluid (CSF) samples were collected prior to the first dose and at day 8 for PK assessments..Drug concentrations were measured by a validated liquid chromatography tandem mass spectrometry method. The steady-state PK parameters of contezolid in plasma and CSF were calculated using a noncompartmental model method. The blood-brain barrier penetration of contezolid was evaluated by the ratio of CSF to plasma drug concentration and exposure.

**Figure:**
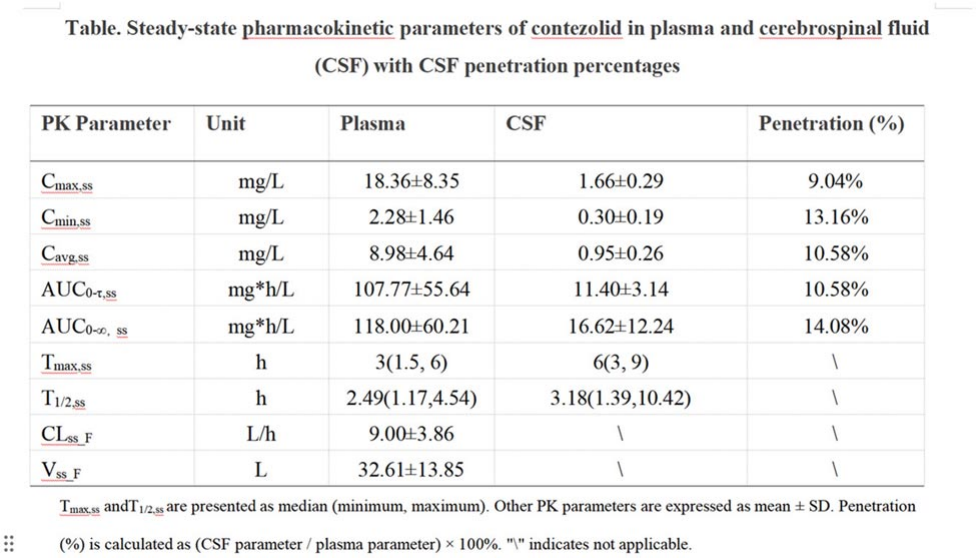

**Results:**

At steady-state, the plasma AUC_0-12,ss_ and AUC_0-∞,ss_ were 107.77±55.64 mg·h/L and 118.00±60.21 mg·h/L, whereas the CSF values were 11.40±3.14 mg·h/L and 16.62±12.24 mg·h/L, with penetration rates of 10.58% and 14.08%, respectively. The T_max,ss_ in plasma and CSF were 3 h (1.5–6 h) and 6 h (3–9 h), and the CSF to plasma drug concentration ratio increased with time, indicating delayed drug entry into the CSF. The T_1/2,ss_ in plasma and CSF were 2.49 h (1.17–4.54 h) and 3.18 h (1.39–10.42 h), suggesting slower elimination in CSF. The plasma clearance (CL_ss_F_) and volume of distribution (V_ss_F_) were 9.00±3.86 L/h and 32.61±13.85 L, respectively.

**Conclusion:**

Contezolid demonstrated moderate CSF penetration, but its CSF concentrations were markedly lower than plasma levels, indicating restricted distribution across the blood-brain barrier.

**Disclosures:**

All Authors: No reported disclosures

